# Characterization of Tuna Gelatin-Based Hydrogels as a Matrix for Drug Delivery

**DOI:** 10.3390/gels8040237

**Published:** 2022-04-12

**Authors:** Carolina Hermida-Merino, David Cabaleiro, Luis Lugo, Jesus Valcarcel, Jose Antonio Vázquez, Ivan Bravo, Alessandro Longo, Georges Salloum-Abou-Jaoude, Eduardo Solano, Carlos Gracia-Fernández, Manuel M. Piñeiro, Daniel Hermida-Merino

**Affiliations:** 1 Departamento de Física Aplicada, CINBIO, Universidade de Vigo, Campus Lagoas-Marcosende, 36310 Vigo, Spain; dacabaleiro@uvigo.es (D.C.); luis.lugo@uvigo.es (L.L.); mmpineiro@uvigo.es (M.M.P.); 2 Grupo de Reciclado y Valorización de Materiales Residuales (REVAL), Instituto de Investigaciones Marinas (IIM-CSIC), Eduardo Cabello 6, 36208 Vigo, Spain; jvalcarcel@iim.csic.es (J.V.); jvazquez@iim.csic.es (J.A.V.); 3 Departamento de Química Física, Facultad de Farmacia, UCLM, 02071 Albacete, Spain; ivan.bravo@uclm.es; 4 ID20, European Synchrotron Radiation Facility (ESRF), 71 Avenue des Martyrs, 38000 Grenoble, France; alessandro.longo@esrf.fr; 5 Istituto per lo Studio dei Materiali Nanostrutturati (ISMN)-CNR, UOS Palermo, Via Ugo La Malfa, 153, 90146 Palermo, Italy; 6 Constellium C-TEC Technology Center, Parc Economique Centr’alp, 725 rue Aristide Bergès, 38341 Voreppe, France; georges.salloum-abou-jaoude@constellium.com; 7 ALBA Synchrotron Light Source, NCD-SWEET Beamline, 08290 Cerdanyola del Valles, Spain; esolano@cells.es; 8 TA Instruments Waters Chromatography, Tres Cantos, 28760 Madrid, Spain; cgracia@tainstruments.com; 9 Netherlands Organisation for Scientific Research (NWO), c/o ESRF BP 220, DUBBLE CRG/ESRF, CEDEX, 38043 Grenoble, France

**Keywords:** rheological hydrogel properties, yellowfin tuna gelatin, secondary structure, drug delivery, Doxorubicin, Crocin

## Abstract

The skin of yellowfin tuna is one of the fishery industry solid residues with the greatest potential to add extra value to its circular economy that remains yet unexploited. Particularly, the high collagen content of fish skin allows generating gelatin by hydrolysis, which is ideal for forming hydrogels due to its biocompatibility and gelling capability. Hydrogels have been used as drug carriers for local administration due to their mechanical properties and drug loading capacity. Herein, novel tuna gelatin hydrogels were designed as drug vehicles with two structurally different antitumoral model compounds such as Doxorubicin and Crocin to be administrated locally in tissues with complex human anatomies after surgical resection. The characterization by gel permeation chromatography (GPC) of purified gelatin confirmed their heterogeneity composition, exhibiting three major bands that correspond to the β and α chains along with high molecular weight species. In addition, the Fourier Transform Infrared (FT-IR) spectra of gelatin probed the secondary structure of the gelatin showing the simultaneous existence of α helix, β sheet, and random coil structures. Morphological studies at different length scales were performed by a multi-technique approach using SAXS/WAXS, AFM and cryo-SEM that revealed the porous network formed by the interaction of gelatin planar aggregates. In addition, the sol-gel transition, as well as the gelation point and the hydrogel strength, were studied using dynamic rheology and differential scanning calorimetry. Likewise, the loading and release profiles followed by UV-visible spectroscopy indicated that the novel gelatin hydrogels improve the drug release of Doxorubicin and Crocin in a sustained fashion, indicating the structure-function importance in the material composition.

## 1. Introduction

Gelatin is a protein obtained by hydrolytic degradation of collagen derived from the skin, tendons, connective tissue and bones of animals, differentiating 27 different types of collagen of which type I is the most widely found [1]. Likewise, the properties of gelatin are mainly influenced by the source and type of collagen that defines its structure based on the triple-helix structure which is formed by three intertwined α-helix chains. The α-helix chains are mainly stabilized by intra- and inter-chain hydrogen bonding that is the product of continuous repeating amino acid sequence Gly-Pro-Y, Gly-Pro-Hyp and Gly-X-Hyp, where X and Y can be any amino acid [1].

The extremely limited solubility of collagen can be overcome by partial hydrolysis and/or denaturation of collagen, rendering a water-soluble functional biopolymer (gelatin) [2] that is extensively used in pharmaceutical, cosmetic and biomedical applications, as well as a food additive and biopolymeric agent [3]. Especially, the gelling and viscoelastic properties of gelatin are important for cosmetic and pharmaceutical applications, and are extensively used in the food industry [1]. Likewise, the extent of gelatin use for industrial applications in recent years has exponentially increased the global demand for gelatin, attaining an estimated gelatin production of 450,000 tons in 2018, with a commercial value of USD 4.52 billion [4].

Industrial fish wastes such as skin, head and bones, which are characterized by a low economic value, were identified as an ideal source to afford gelatin whilst strengthening the circular economy of the fish industry by converting an abundant fish processing discard into a value-added product [5]. In addition, the generation at the industrial scale of the fish skin endorses it as an alternative source to mammalian gelatin due to the increasing social and religious concerns about its use. However, the characterization of the physicochemical, structural and rheological properties of fish gelatin is still needed to extend its industrial applications [6].

Among fish commercial species, the potential of tuna skin gelatin stands out due to its high economic value and extensive international trade. The amount of world tuna production in 2016 was approximately 6.6 Mt and has increased by 2% since 2017 (FAO 2019). In particular, yellowfin tuna (*Thunnus albacares*) is one of the main species of tuna caught in the Indian Ocean Fisheries Management Area (FMA) 573, contributing to 35.83% of total tuna production in 2013 [7]. In addition, tuna gelatin has a higher content of key amino acids such as proline and hydroxyproline content compared to cold-water fish species, endowing it with structural strength similar to its mammalian counterpart [8,9,10].

Gelatin-based hydrogels have also gained significant attention in the field of tissue engineering due to their biocompatibility, biodegradability and well accessible cell adhesion, promoting bioactive Arg-Gly-Asp (RGD) domains in their peptide chain structure that mimic the extracellular matrix (ECM) [1] which makes it a highly desirable biomaterial for medical applications [2]. In addition, hydrogels formed by gelatin are attractive materials for drug delivery systems, particularly for local administrations due to their characteristic mechanical properties as well as low levels of immunogenicity and cytotoxicity [11,12].

**Figure 1:**
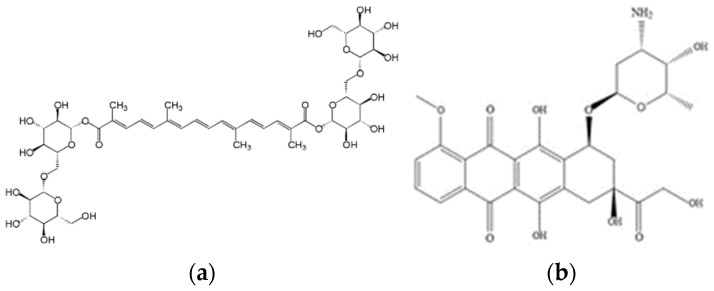
Scheme of the molecular structures of (**a**) Crocin and (**b**) Doxorubicin.

Likewise, the development of tuna skin gelatin hydrogels has the potential to design carrier vehicles for antitumoral therapeutic agents for local administration in complex anatomical areas after cancer resection [13] to minimize harmful side effects in healthy cell tissues as high doses of antitumoral are required to be systemically administered to exert any therapeutic benefit [14]. The loading/releasing capacity of the tuna gelatin hydrogels was assessed with two structural different antitumoral compounds, Doxorubicin (DOX) and Crocin (Figure 1), to understand the role of the intermolecular interactions.

DOX is a multiring planar molecule, which can provide appropriate binding sites through π-π and hydrogen bonding interactions on the transport of other guest molecules. In addition, a substantial drug release may be expected due to its amphiphilic nature with aqueous solubility [15,16,17].

Crocin is a carotenoid present in saffron that possesses various health-promoting properties, including antioxidant, memory enhancer, antidepressant, anxiolytic and aphrodisiac [18] and has been reported to manifest antitumoral activity [18,19,20]. Crocin is a flexible molecule featuring multiple functional groups which are characterized by a hydrophilic character that makes it an attractive candidate for drug development. Although the strength of each hydrogen bond has not yet been considered, likely, the formation of excessive amounts of hydrogen bonding along the ligands between Crocin and gelatin will lead to a more stable binding mode [20].

Previously, tuna skin gelatin was used to generate solid capsules [21] for drug delivery applications that were proved to be superior to the commercial capsules as well as the formation of nanogels derived from fish gelatin methacryloyl [22] to load Doxorubicin due to the featured excellent medical properties such as antioxidant, anticancer and antimicrobial activity [12]. Herein, the structure–property relationship of novel hydrogels based on tuna skin gelatin (*T. albacares*) has been characterized to exploit them as drug carrier vehicles. Likewise, the physicochemical, structural and rheological properties of the generated tuna hydrogels were evaluated to understand the load and release mechanism of the loaded therapeutic agents.

## 2. Results and Discussion

Gelatin hydrogels formed at a fixed gelatin concentration (25% gelatin) were loaded with the selected model antitumoral (DOX and Crocin) due to the featured promising mechanical properties for the drug storage and subsequent delivery, to understand the role of the drug structure on the loading and releasing mechanism for designed targeted drug delivery systems (Table 1).

**Figure 2:**
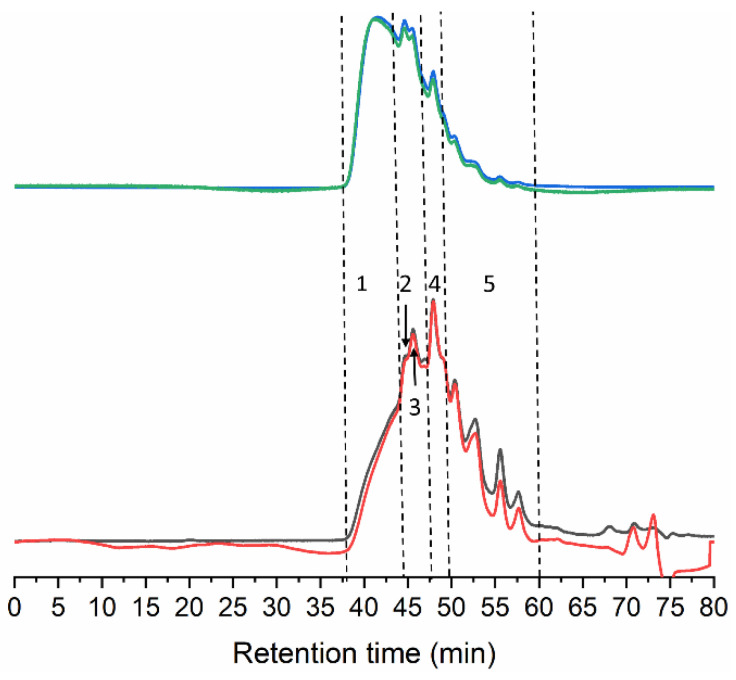
GPC eluograms of gelatin extracted from the skin of tuna. Blue line: right angle light scattering; green line: low angle light scattering; red line: refractive index; black line: ultraviolet (280 nm).

The gelatin chain structure obtained by the extraction method from tuna collagen was characterized by GPC and TGA. The GPC eluogram of tuna gelatin (Dry GE) exhibits several overlapping polymeric peaks, starting at 38 min with the elution of high Mw species, up to around 60 min when light scattering signals return to baseline (Figure 2) which is in agreement with skin gelatin from other fish species obtained using an identical extraction process [23,24,25,26].

The Mw distribution consists of five different peaks/regions (Table 2). The best-resolved peak at 48.3 min likely corresponds to α-chains, as the estimated Mw of 117.0 kDa falls within its typical Mw range (80–125) [27]. In addition, two extra peaks were observed at 156.5 and 228.0 kDa. The latter indicates the presence of dimers of α-chains covalently linked (β-chains) that usually feature Mw between 160–250 kDa. The fraction of 156.5 kDa might correspond to partially degraded β-chains or α-chains linked to peptide fragments.

The Mw for the regions at the extremes of the distribution cannot be accurately determined, however, the first eluting fraction easily surpasses 300 kDa, and probably includes trimers (γ-chains) and other higher molecular weight (HMw) aggregates resulting from the hydrolysis of collagen fibrils. The stretch eluted between 50 and 60 min corresponds to degraded peptides below 100 kDa (Figure 2 and Table 2).

**Figure 3:**
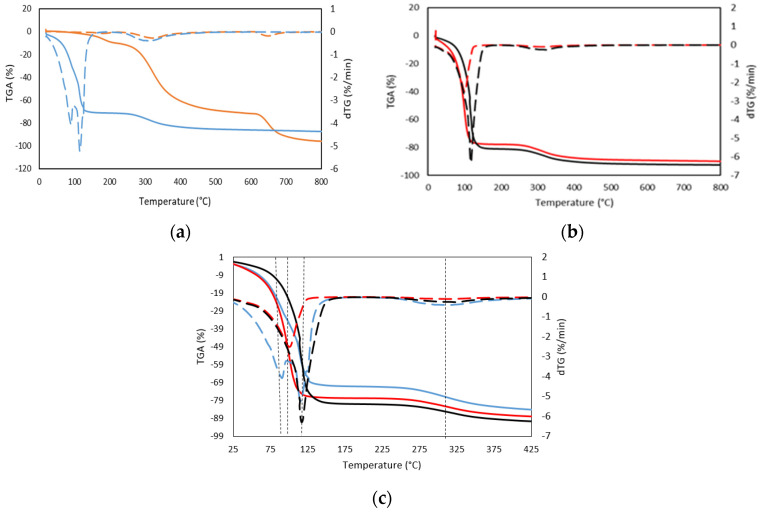
TGA thermogram (solid line, weight loss), and dTGA thermogram (dashed line, differential weight loss) obtained from TG curves of (**a**) (**-**) Dry GE (**-**) 25GE, (**b**) (**-**) 25GE/DOX (**-**) 25GE/Crocin, and (**c**) comparative plot of TGA thermogram (solid line, weight loss), and dTGA thermogram (dashed line, differential weight loss) of 25GE, 25GE/DOX and 25GE/Crocin.

Three different stages were mainly identified by TGA and its corresponding dTGA, in both dry gelatin and gelatin hydrogels (Figure 3a,b, Table 3 and Table 4). In the first degradation stage, a weight loss of 11% occurred much earlier for the dry gelatin than for the gelatin hydrogels counterparts, however, the higher exhibited maximum peak (182 °C) is likely due to the degradation or decomposition of polysaccharide/protein components of the gelatin and lower molecular weight glycerol compounds [28], together with the evaporation of the adsorbed and bound water present in the dry gelatin specimen due to its hygroscopic character [29]. Likewise, the gelatin hydrogel presents a first degradation maximum step in the temperature range from 90 °C to 117 °C, with a weight variation of the order of 71% of weight, which is related to the different forms of association of water with the gelatin structure [30]. In particular, the first stage of weight loss is primarily associated with the loss of free and bound water adsorbed on the gelatin. The free (adsorbed) water is in the form of individual clusters and is removed from the material at a lower temperature while the desorption of structural bound water appears to occur at higher temperatures.

On the other hand, the decomposition temperatures of drug-loaded gelatin hydrogels were found similar to the gelatin hydrogel (see Figure 3c). However, the observed first biphasic transition for the tuna gelatin hydrogel, with maxima at 90 °C and 117 °C and with a weight loss in the first stage of 70%, suggests probing different water environments with diverse interactions with the gelatin. Furthermore, the first transition of drug-loaded hydrogels appeared as only one transition that corresponds to the individual maximum of the tuna gelatin hydrogel, 101 °C and 117 °C for 25GE/DOX and 25GE/Crocin, respectively, and with a weight loss of around 80% which suggests that the drug interactions with the gelatin promote different configurations with water affinities. Likewise, the slightly lower first-stage weight loss exhibited by the fish gelatin hydrogel as well as the observed changes between the dTGA endothermic peaks of the loaded and 25GE hydrogels suggests less desorption of water from the hydrogen-bonded matrix of the tuna hydrogel in comparison with hydrogels loaded with drugs, and thus the miscibility and interaction between the gelatin and the drug.

The second stage, corresponding to the greatest weight loss for the dry gelatin, occurred above 300 °C (Table 3). This has been associated with the breakdown of the gelatin peptide bonds due to the degradation of the low molecular weight protein fraction and structurally bound water [31], which was higher than the value of gelatin hydrogels due to the higher concentration of gelatin.

Similarly, the next weight-loss stage (12%) of the drug-loaded hydrogels occurred at a slighter lower temperature (300 °C) in comparison with the gelatin hydrogel (310 °C and loss of 16%) which is likely related to the degradation of the larger or associated protein fragments. The temperature difference between the gelatin hydrogel and the drug-loaded tuna hydrogels reinforces the previously observed indications about the interaction between the drug and gelatin molecules [32,33,34]. The last exhibited degradation stage at 600 °C has been attributed to the thermal decomposition of the gelatin network due to the formation of covalent bonds in the gelatin network that leads to higher thermal stability. Consequently, the thermal stability of the gelatin composition found by GPC during the preparation (65 °C) of the hydrogel is ensured, as well as the stability of the hydrogel at body temperature, which is key to avoiding a rapid initial burst under the conditions of administration.

The interaction between gelatin and drugs through likely hydrogen bonds as well as its chemoselectivity have been analyzed by probing the secondary structure of gelatin by FTIR (α-helix and β-sheet). Doxorubicin and Crocin, selected as model drugs by their structural differences, are expected to interact through both van der Waals and hydrogen bonding interactions, depending on their functional groups, which could potentially affect the secondary structure of gelatin.

Gelatin with higher α-chain content has been reported to feature promising functional properties, including gel strength [35] and gel-forming ability. In particular, the conversion of α-helix into the random coil controls the gel transition whilst the β-sheet contributes to increasing the gel strength [36]. The FTIR amide bands are essential for determining the secondary structures as they form part of the polypeptide chain backbone, and their association by hydrogen-bonding interactions generates the different structural conformations (α-helix, β-sheet/turn and random coil) [37]. The typical gelatin FTIR spectrum contains the vibration bands of the corresponding multiple components extracted from the gelatin with low intensities for the amide band I and II, while the amide band III is almost non-existent which complicates its interpretation. Besides, the changes in the gelatin FTIR bands related to the secondary structure (Amide-I and Amide-II) of the hydrogels could also have arisen from the formation of hydrogen bonds between molecules with water [38]. Furthermore, the aqueous dissolution of gelatin could exchange the amide nitrogen hydrogen for water molecules [39], although the carbonyl groups tend to be placed in hydrophobic pockets.

However, the amide I band is still useful to identify the secondary structure of proteins by FTIR [40] as it is sensitive to the hydrogen bonding interactions and the conformation of the protein structures [41]. Likewise, the FTIR will probe the expected changes due to the denaturation of collagen to gelatin that results mainly in the loss of the triple helix.

**Figure 4:**
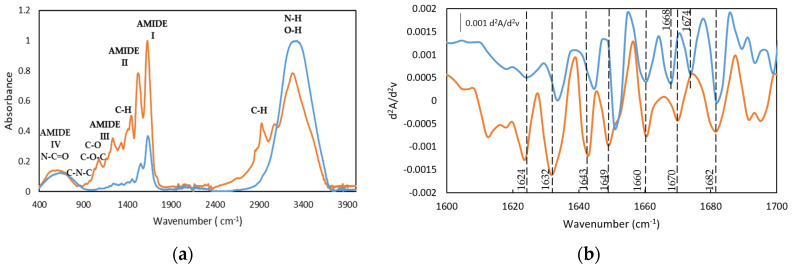
FTIR spectra (**a**) and spectra of the second derivative (differential FTIR spectra) in the absorption region of amide I by FDD method (**b**), of (**-**) Dry Tuna (**-**) Tuna gelatin hydrogel.

Similarly to other fish gelatins, the FTIR spectra of both gelatin and the gelatin hydrogel are characterized by the main amide bands, such as the Amide I band in the frequency range of 1640–1630 cm^−1^, Amide II in the range of frequency of 1560–1540 cm^−1^, and the band in the range of frequency of 1238–1244 cm^−1^ assigned to Amide III (Figure 4a and the second derivative method Figure 4b and Table S1) [23,42,43,44,45].

The bands centered at 1631 and 1634 cm^−1^, particularly significant for the dry GE, can be ascribed to β segments [43,46] although they have also been associated with the hydroxyproline content in the collagen fibers as the 1628–1633 cm^−1^ region is characteristic of the absorption of imine carbonyls [47]. Furthermore, the band at 1682 cm^−1^ is related to β chains and the band in the spectral region between 1660 and 1690 cm^−1^ are due to β turns [46]. In addition, the presence of the bands between 1642 and 1624 cm^−1^ confirmed the existence of the β sheets whilst the band located at 1649 cm^−1^ probed the appearance of the random coil. In addition, the relative intensity of the second derivative bands suggests the predominance of the triple helix and β turn in the gelatin hydrogel structure.

**Figure 5:**
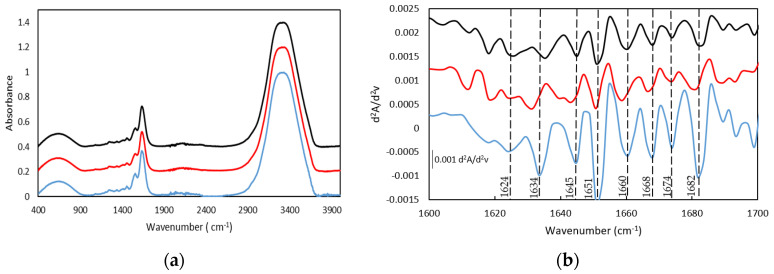
FTIR spectra (**a**) and Spectra of the second derivative (differential FTIR spectra) in the absorption region of amide I by FDD method (**b**), of (**-**) 25GE, (**-**) 25GE/DOX and (**-**) 25GE/Crocin.

Similarly, the FTIR drug-loaded gelatin hydrogels spectra contain the vibration bands of the gelatin secondary structure (Figure 5 and Table 5). In particular, the Amide I band is constituted by the presence of the β-sheet, at 1631 cm^−1^ and 1635 cm^−1^ as well as a band at 1643 cm^−1^ and 1645 cm^−1^ due to the coexistence of the random coil conformation. Likewise, the vibration bands at 1651/1659 cm^−1^ and 1651/1656 cm^−1^ proved the presence of the α-helical structure for both drug-loaded hydrogels, 25GE/DOX and 25GE/Crocin, respectively. Moreover, the β-turn was also found in the drug-loaded hydrogels with bands at 1681cm^−1^ and 1684 cm^−1^, suggesting a similar structure for both gelatin hydrogel and drug-loaded hydrogels.

However, the increase of the relative intensity of the random coil band and the observed shift of the Amide I band towards higher wavenumbers for the drug-loaded hydrogels (Figure 5b), as well as the absence of the main drug bands in the drug-loaded hydrogels, could indicate the interaction of the drug agents and the gelatin (for instance, the 1730 cm^−1^ band of the C=O ketone group of the DOX drug as well as the Amide IV, V and VI bands of the 25GE disappeared in the 25GE/DOX and 25GE/Crocin hydrogels).

**Figure 6:**
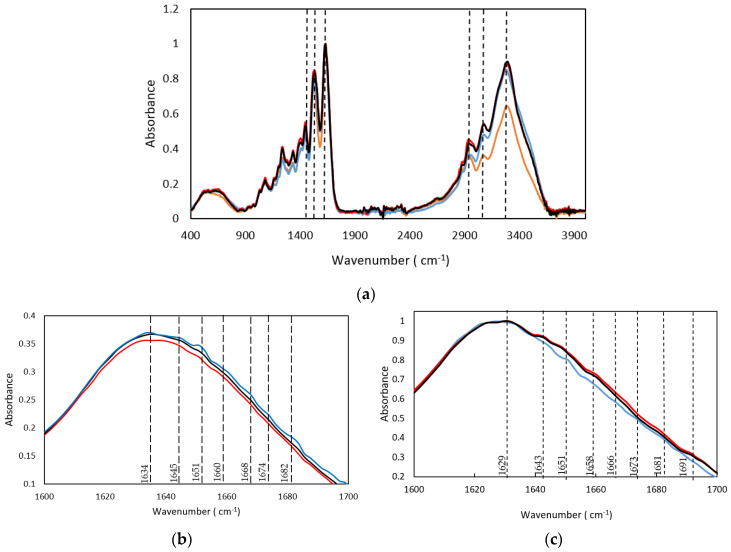
(**a**) FTIR spectra at 50 °C, (**b**) FTIR spectra at room temperature and (**c**) FTIR spectra at 50 °C; in the 1600–1700 cm^−1^ region of (**-**) Dry GE, (**-**) 25GE, (**-**) 25GE/DOX and (**-**) 25GE/Crocin.

In addition, FTIR spectroscopy of the hydrogels at 50 °C was conducted to understand the structural transitions that generate the physical crosslinks during the reversible gelation. The gelatin FTIR bands followed a shift in temperature related to changes in the triple helix structure and β structures (Figure 6 and Figure S1 and Table S2).

Furthermore, the 3290–3280cm^−1^ region is dominated by the NH-bond stretch mode of hydrogen-bonded amide groups. The absorption is polarized parallel to the NH bond, which is parallel to the helix axis in α-helical structures and perpendicular to the polypeptide chain in β-sheets. The amide A band associated with NH stretch vibration is sensible to the existence of hydrogen bonds as typically a free N-H stretching vibration occurs in the range of 3400–3440 cm^−1^ whilst the N-H group of a peptide is involved in a hydrogen bond, where the resonance shifts to lower frequencies [48].

However, as temperature increases, FTIR spectra reveal a decrease in OH stretching; this is attributed to acceleration of the dehydration reaction in gelatin, suggesting a decrease in polar functional groups with increasing temperature [49].

The shift to lower frequencies of the amide A band observed in the spectra of the hydrogels at 50 °C compared to the room temperature proves the involvement of the peptide NH group in a hydrogen bond [50], indicating a greater disorder from a helical structure to a random coil structure as well as with the loss of the triple helix state [35].

Likewise, the amide I band associated with the C=O stretching vibrations of the amide groups coupled to the in-plane NH bending and CN stretching modes [40] also probes the hydrogen bond involving the C=O and NH moieties. The FTIR spectra of the dry gelatin showed that the amide I band centered at 1631cm^−1^ which is indicative of the β sheet structure remained constant during the thermal treatment at 50 °C (Figure 6a). However, a small frequency shift of the amide I band (1628–1630 cm^−1^) was observed for the hydrogels (25GE, 25GE/DOX and 25GE/Crocin, Figure 6c) which are characteristic of intermolecular hydrogen bonding [51], suggesting a strong interaction within the gel network.

Likewise, the variable temperature FTIR probed the thermal dynamic behavior of the secondary structures and functional groups of gelatin hydrogels that determine the formation of the hydrogels during the reversible thermal cycle.

**Figure 7:**
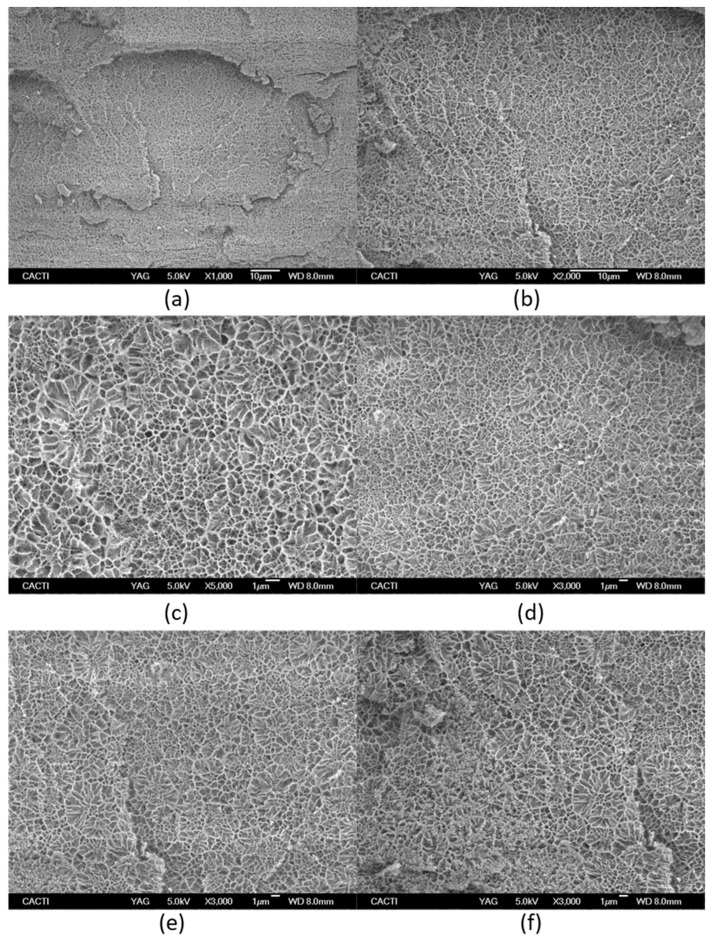

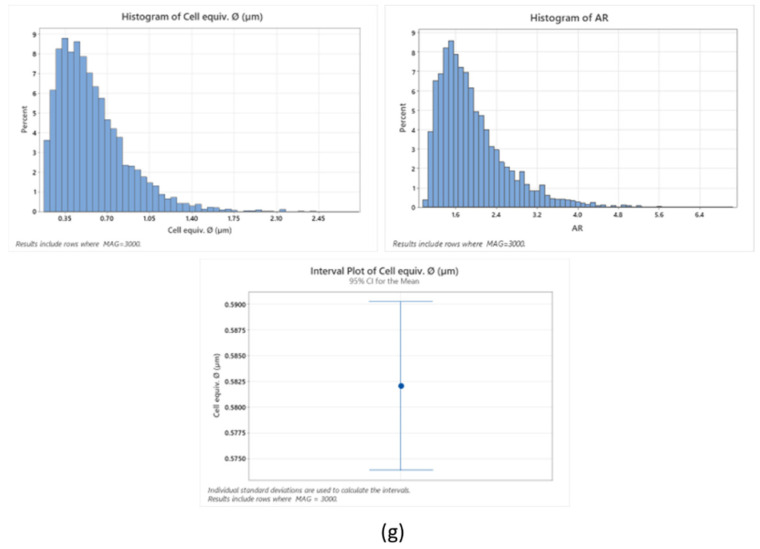
Cryo-scanning electron microscopy of Tuna gelatin hydrogel at different magnifications: (**a**) ×1000, (**b**) ×2000, (**c**) ×5000, (**d**–**f**) ×3000**.** (**g**) Statistical analysis of cell size-distributions and cell aspect-ratio distributions obtained from the three micrographs captured at X3000 magnification (average of three results).

In addition, morphological analysis of the hydrogel network was performed by cryo-SEM (Figure 7) as the hydrogel structure is an important prerequisite to be applied as a drug carrier. The interconnected pores typically found in the morphology of the hydrogel are crucial to controlling the drug release rate. Similarly, the degree of aggregation and phase separation determine the rheological properties that are associated with the releasing drug profile from the hydrogel network.

However, cryo-SEM commonly studies the collapsed hydrogel structure due to the sample preparation, in particular after etching where etching involves semi-drying of the hydrogel and thus, experimental artefacts limit the morphological analysis [52].

The microstructure of tuna gelatin hydrogel was probed at several magnifications (Figure 7) where darker areas in the micrographs correspond to amorphous water which was not sublimated during the sample preparation process, whereas lighter objects are associated with gelatin structures after etching.

The tuna gelatin hydrogel morphology was characterized by a sponge or coral-like structure, with a large number of interconnected small pores fairly homogenous both in size and space ratio (Figure 7g, around 0.6 µm) and denser networks that typically yield good gelling properties [53] whilst larger pores were also found that are likely correlated with low aggregation of peptide chains during gelation [54].

The morphology revealed by cryo-SEM is in good agreement with the presence of a significant proportion of high molecular weight as well as reflecting its large molecular weight distribution profile (Figure 2). Besides, a controlled and enhance sustained drug release is envisaged from the small size of the obtained pores as well as the presence of fibrous like structures within the pores [55].

**Figure 8:**
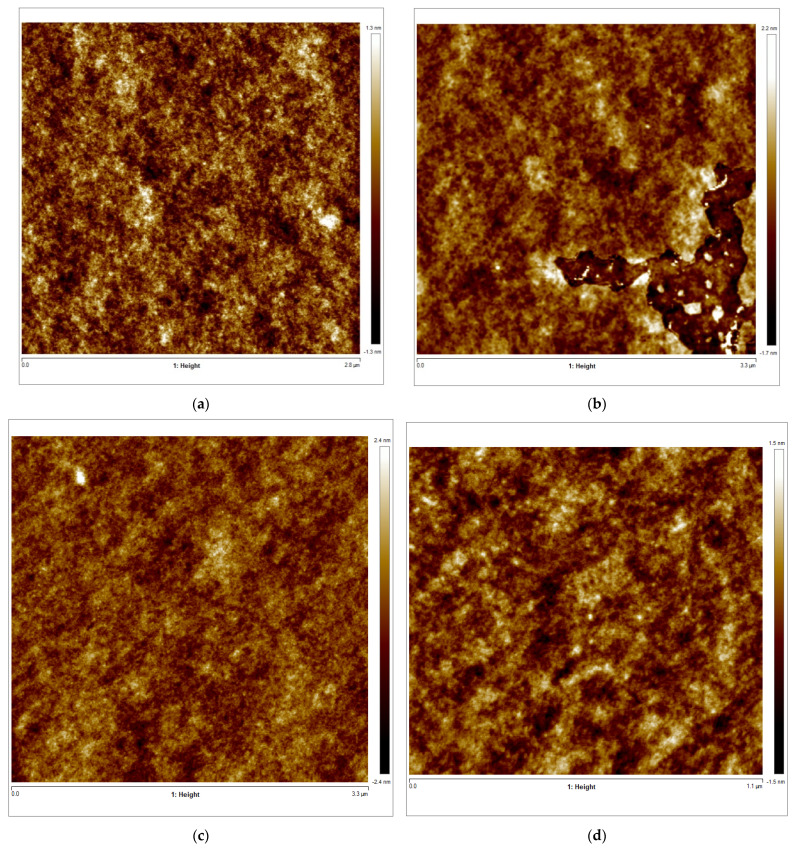

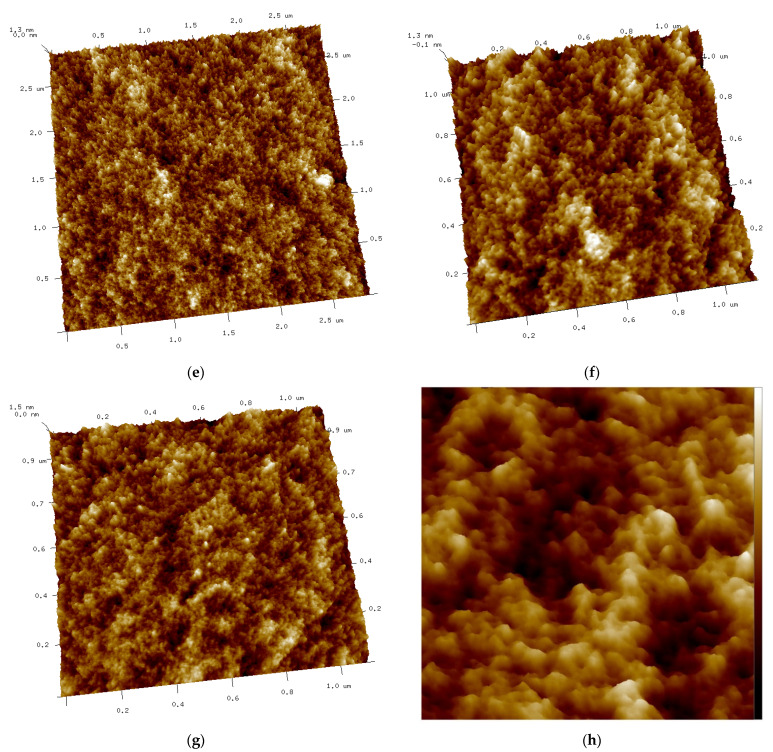
(**a**–**d**) AFM 2D images of Tuna gelatin hydrogel. (**a**) Zone 1 at a magnification of 2.8 × 2.8 µm^2^, (**b**) Zone 2 at a magnification of 3.3 × 3.3 µm^2^, (**c**) Zone 3 at a magnification of 3.3 × 3.3 µm^2^, (**d**) Zone 3 at a magnification of 1.1 × 1.1 µm^2^. (**e**–**g**) AFM 3D images of Tuna gelatin hydrogel. (**e**) Zone 1 at 1.2 nm, (**f**) Zone 2 at 1.3 nm, (**g**) Zone 3 at 1.5 nm. (**h**) Zoom images 3D in zone 1 with magnifications 120–120 nm^2^.

In addition, the microstructure of the tuna gelatin hydrogel was studied by AFM, exhibiting a rough surface with greater variability of protrusions (yellow areas) and with separate aggregates with relatively clear edges in Zone 1 and Zone 2 (Figure 8a,b). The gelatin aggregates featured a spherical irregular like structure with a polydisperse diameter size. In contrast, a continuous surface with identical height (brown color) and some protrusions (yellow color) but without significant cavities were found in Zone 3 (Figure 8c,d) that featured a coacervated structure without a defined geometric shape, similar to that previously observed [56].

Furthermore, the surface structure resolved in the AFM micrographs with the highest magnification (1.1 × 1.1 µm^2^) allowed the quantitative analysis of the topography which confirmed the persistence of the nanostructure at a lower length scale (Table 6). Likewise, the trabecular structure obtained by AFM in the hydrogel is in good agreement with the porous network observed by cryo-SEM on the partially collapsed hydrogel network (Figure 7).

**Figure 9:**
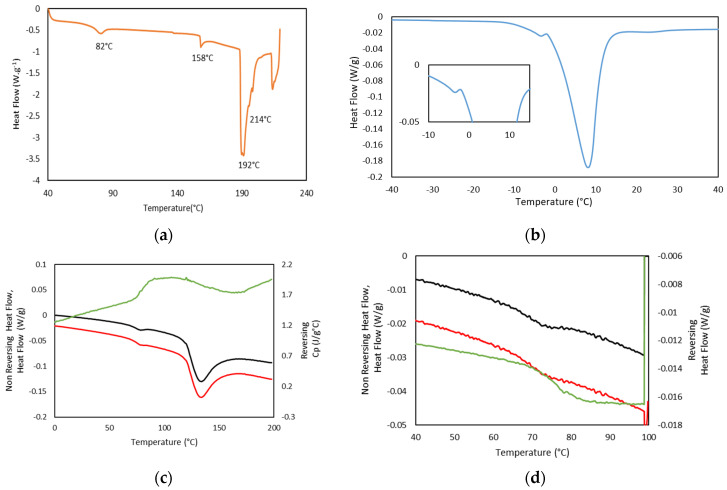
(**a**) DSC thermogram of Dry GE, heating ramp at 5 °C·min^−1^, (**b**) DSC Thermogram of 25GE, heating ramp at 1 °C·min^−1^, (**c**) MDSC Dry GE in the Rev Cp (green color), Non Reversing Heat Flow (black color) and Heat Flow (red color), heating ramp, at 1 °C/min, (**d**) MDSC Dry GE in the Rev Heat Flow (green color), Non Reversing Heat Flow (black color) and Heat Flow (red color), second heating ramp, at 0.5 °C·min^−1^.

In addition, thermal analysis of the formed hydrogels, as well as Dry Ge (Dry GE, 25GE, 25GE/DOX and 25GE/Crocin), were studied by standard differential scanning calorimetry (DSC) at different cooling/heating rates and modulated DSC (MDSC) to understand the weak thermal transitions that firstly allow the gelatin to solubilize on heating and subsequently form the hydrogel network on cooling. The first heating ramp of the dry tuna gelatin DSC thermogram (Figure 9a) shows a second-order thermal event between 65 and 75 °C that could be related to the glass transition associated with its amorphous part [57] which is followed by four endothermic peaks at about 82 °C, 158 °C, 192 °C and 214 °C. The first endothermic transition at 82 °C could be associated with kinetic trapped structures during the gelatin preparation whereas the following endothermic event at 158 °C may be due to the evaporation of the chemically bound water content remaining in the drying process and the water adsorption during storage or conformational changes in the protein structure [30,58] as observed by TGA (Figure 3). Furthermore, the thermal events at about 200 °C may be associated with the gelatin degradation process with loss of amino acids as previously revealed by TGA (Figure 3). The thermal reversibility or partially irreversible denaturation of gelatin was assessed by a series of thermal cycles with heating and cooling ramps. The reversibility of the thermal event that appeared at 65 °C confirmed that no degradation nor evaporation occurs from −80 to 100 °C (Figure S2a), but a partially reversible denaturation could not be discarded. In addition, the DSC of the 25GE hydrogel revealed the differences in the melting of water (Figure 9b is the first heating ramp of the full DSC cycle reported in Figure S2b) that confirmed the diverse gelatin/water environments observed by TGA.

MDSC of Dry Tuna gelatin (Figure 9c,d as well as Figure S3) were also performed due to their higher sensitivity to confirm the presence of the glass transition at 68.9 °C (Table 7) that was evident in the reversing heat flow curve together with a water evaporation event and or enthalpic recovery due to the presence of an endothermic peak in the non-reversible thermogram (Figure 9d). Similar thermal behavior was also previously [58] observed in gelatin extracted from yellowfin tuna skin which was attributed to kinetic extensive structural rearrangements.

Likewise, MDSC experiments involving several thermal cycles on the same specimen were conducted to verify the reversibility of the thermal transitions (Figure S3b). The identification of the glass transition is key to designing the hydrogels as it is related to the movement of the molecular segments of the disordered amorphous part, which is associated with the chain stiffness, molecular weight or architecture/type of isomers.

**Figure 10:**
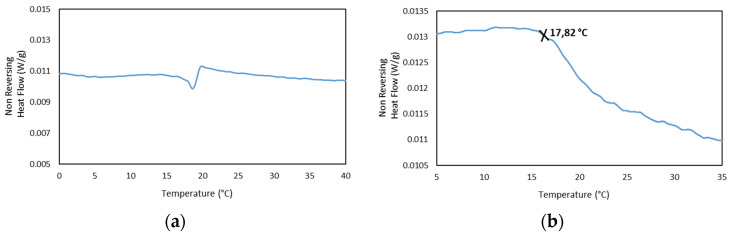
MDSC of 25GE thermogram of the (**a**,**b**) Non Reversing Heat Flow of the cooling ramp at 0.50 °C/min.

In addition, the MDSC Thermograms of the 25GE hydrogel proved the presence of the glass transition at ca. −65 °C for 25GE hydrogel (Table 7). The reduction in the glass transition temperature of the hydrogel in comparison with the dry Tuna gelatin is associated with the lubrication of gelatin by water molecules [58] due to its bridging action of the macromolecular network [58]. Furthermore, the MDSC of the 25GE hydrogel revealed the sol/gel transition in the ca. 17.8 °C to 25 °C temperature range (Figure 10).

**Figure 11:**
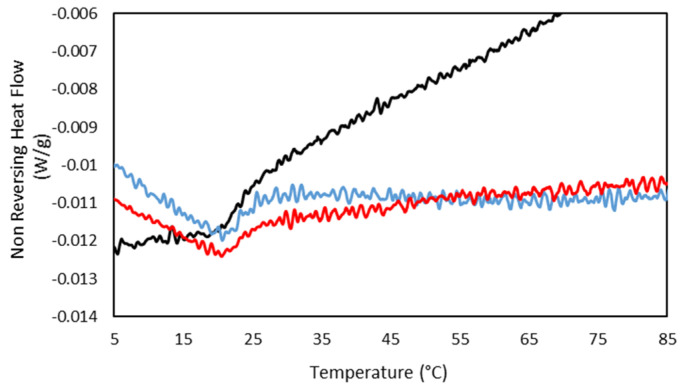
MDSC thermograms of the Non Reversing Heating scan of (**-**) 25GE, (**-**) 25GE/DOX and (**-**) 25GE/Crocin at 0.5 °C·min^−1^.

In addition, the concurrence of the sol/gel transition found by MDSC at the same temperature for the drug-loaded hydrogels compared to the gelatin hydrogel (Figure 11) shows the persistence of the hydrogel network, indicating the incorporation of the drug to the gelatin network and in agreement with the FTIR.

The rheological properties of gelatin hydrogels were examined to assess the mechanical performance of the hydrogels for drug delivery applications by evaluating their critical deformation and storage capability.

**Figure 12:**
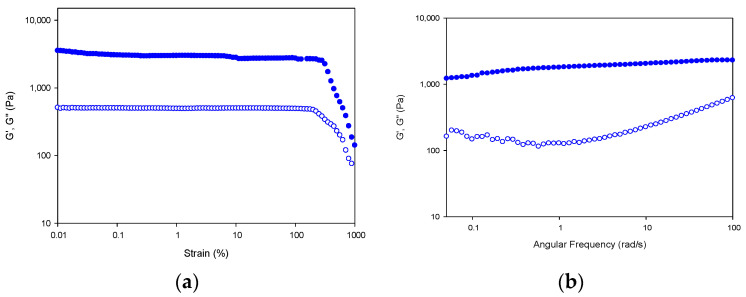
Strain and frequency sweep for Tuna gelatin hydrogel. Storage (G′; filled symbols) and loss moduli (G″; hollow symbols) depicted versus strain (**a**) and angular frequency (**b**) at 20 °C.

First, the stability of the hydrogel network in the linear viscoelastic region (LVR) of the fibrillar lattice was probed by subjecting the hydrogel to strain sweeps at 20 °C, in which the strain amplitude was varied from 0.1 to 1000% at a constant frequency of 10 rad s^−1^ (Figure 12a).

In addition, the resistance of the gel network at 20 °C (at the sol/gel transition) can be assessed by studying the frequency dependence of the viscoelastic moduli (Figure 12b). The hydrogel network shows a gel-like behavior as indicated by the superior storage modulus over the loss modulus (G′ > G″) with a parallel like profile in the frequency range under study [59]. The high values for storage and loss modulus of the tuna gelatin hydrogel indicate the presence of a high concentration of its helical structures [5].

**Figure 13:**
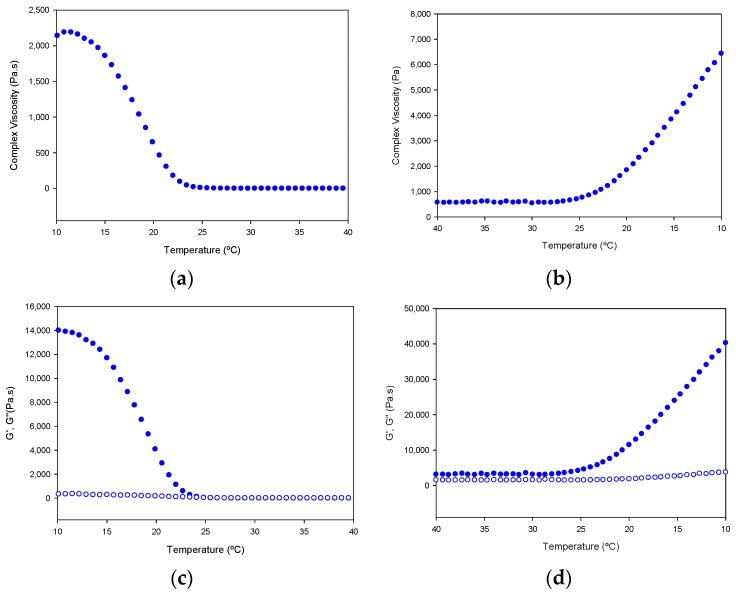
Variation of complex viscosity (**a**,**b**); and storage modulus (G′, filled circle) and loss modulus (G′′, empty circle) (**c**,**d**); with the temperature of Tuna gelatin hydrogel. Heating ramp (left column) from 10 to 40 °C, cooling ramp (right column) from 40 to 10 °C.

In addition, the tuna gelatin hydrogel features a highly complex viscosity (Figure 13), similar to previously discussed fish gelatins [23,24,25] that is typical of a strong gel matrix in agreement with the previous cryo-SEM analyses (Figure 7). Generally, the higher viscosity of gelatin at steady conditions permits it to maintain the integrity of the gel formulation whereas the change of viscosity produced at the sol/gel transition upon shearing makes it suitable for preparing complex structures with good shape printability upon cooling [5].

Furthermore, the gelation kinetics of the tuna gelatin hydrogel were determined by monitoring the complex, loss and storage moduli as well as the viscosity on heating in the critical gelation temperature range as revealed by MDSC (10–40 °C) and its subsequent reversibility upon cooling (40–10 °C, Figure 13). A sharp transition in complex viscosity occurs in the thermal interval between 18 °C and 25 °C (Figure 13a,b) as well as the storage and loss moduli (Figure 13c,d) during the heating ramp, which is indicative of the disruption of the hydrogel elastic network and in agreement with the sol/gel transition observed around for 25GE hydrogel by MDSC that is similar to previously reported in the literature for other fish gelatin [5,24,25].

The shift in the transition observed in the cooling ramp, for both the storage and loss moduli as well as the complex viscosity suggest the process is not fully reversible, however, the partial evaporation of the hydrogel cannot be discarded during the sample measurement.

**Figure 14:**
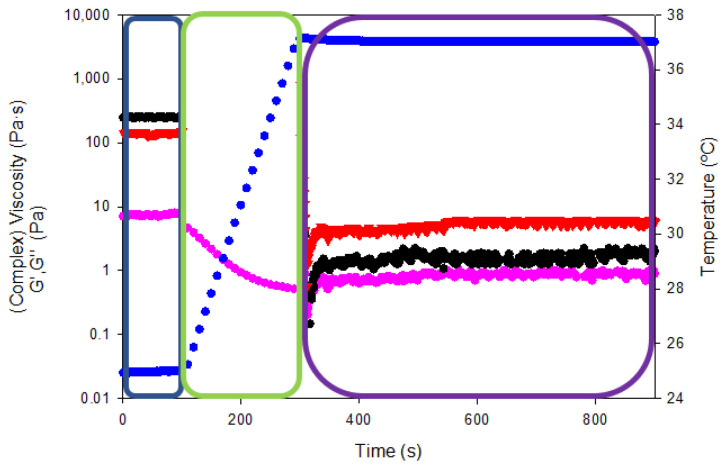
Rheological curves as a function of temperature and intra-articular injection stage (shear rate). Viscosity/complex viscosity (pink color), temperature (blue color), G′ (black color) and G″ (red color) for tuna gelatin hydrogel.

In addition, the so-called “syringe test” rheological analysis was conducted to evaluate the mechanical performance of the gelatin hydrogels for the pharmaceutical administration by a hypothetical injection into the body with a syringe of a specific diameter (0.9 mm) (Figure 14). The viscoelastic properties of hydrogels were measured on three steps with different shear rates and temperatures to mimic the storage capability prior to the injection into the body and the subsequent mechanical performance within the human body.

The resting state of the gelatin hydrogel in the syringe at room temperature (blue box in Figure 14) was assessed by applying an oscillatory sweep of constant 0.1% strain (within the linear viscoelastic range according to oscillatory strain sweeps reported in Figure 12a) and frequency (1Hz). The complex viscosity of the gelatin hydrogel at room temperature was 7.4 ± 0.3 Pa·s while the storage and loss moduli were 247.4 ± 2.6 and 143.3 ± 5.4 Pa·s, respectively. Thereafter, the shear rate was increased to 100 s^−1^ and the temperature was gradually raised to 37 °C to simulate the injection of the hydrogel into the human body (using a 0.9 mm gauge needle, green box Figure 14). The viscosity reduction of the tuna gelatin hydrogel by the end of the second step to below 0.5 Pa·s proved its shear rate and temperature dependence that is key to ensure adequate intra-articular administration of gel-like systems through a specific needle.

Finally, the persistence of the viscoelastic properties within the human body was tested (purple box in Figure 14) by a constant oscillatory sweep at a constant 0.1% strain and a frequency of 1 Hz at a temperature of 37 °C (physiological conditions) yielding a complex viscosity of 0.94 ± 0.09 Pa·s as well as storage and loss moduli of 2.1 ± 0.2 and 6.0 ± 0. Pa·s, respectively. The viscous modulus (G″) predominates over the storage modulus (G′) at temperature body conditions which manifest even if the material has a certain elastic structure, fluid-like behavior that predominates at rest state. Therefore, the rheological test showed that the tuna hydrogel gelatin presented suitable viscosities for intra-articular injection through a 0.9 mm diameter needle.

**Figure 15:**
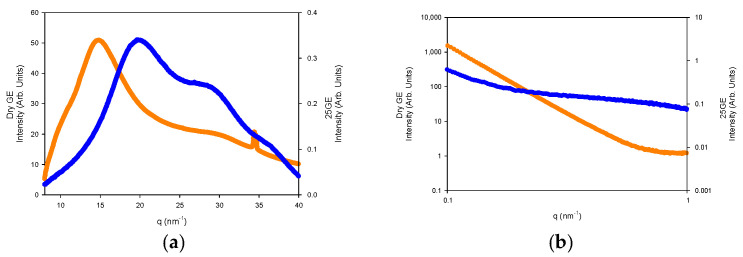
(**a**) WAXS and (**b**) SAXS of Dry GE (Orange line) and 25GE (blue line) at 5 °C·min^−1^ at 25 °C.

The nanostructure of the generated gelatin hydrogels was also probed by small-angle X-ray scattering (SAXS) and wide-angle WAXS measurements (Figure 15a,b).

The WAXS profiles of the dry gelatin indicate the presence of the α-helices or the triple helix (5.8 nm^−1^ and 22 nm^−1^) within the gelatin aggregates by the presence over the amorphous halo (ca 14 nm^−1^) that define the domain distance of the triple helix around 11–12.6 Å as well as the adjacent amino acid distance with a repeat distance of 2.9 Å (Figure 15a) [60]. However, the lack of an oriented SAXS pattern (Figure 15b) confirms the random distribution of the gelatin aggregates and the absence of collagen fibrils within the hydrogel network [61].

**Figure 16:**
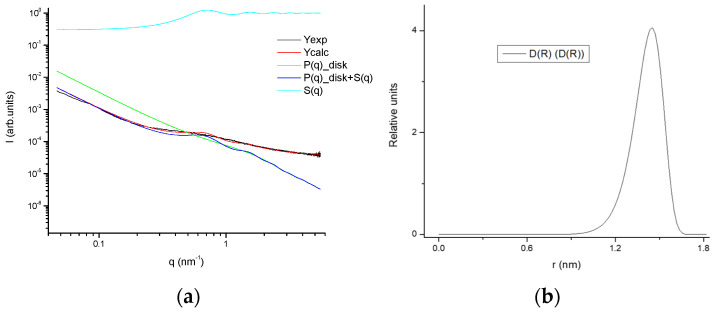
(**a**) Experimental SAXS profile as well as fitted together with the model contributions; (**b**) size distribution of the aggregates of the 25GE hydrogel.

The fitting of the SAXS profiles (Figure 16) suggests the occurrence of gelatin planar aggregates of a fairly monodisperse size that are closely interacting forming a network that at a higher length scale would generate the porous network. Furthermore, the SAXS of the dry GE followed a behavior typical of well-defined sharp interfaces of bigger objects as well as the lack of the quarter staggered order of the packed fibrils of collagen that confirms the disruption of the collagen hierarchical order by the extraction methodology.

**Figure 17:**
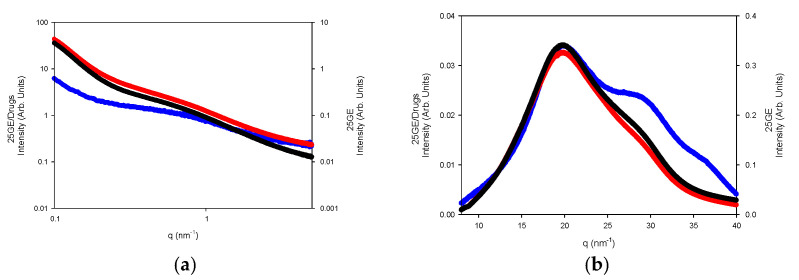
(**a**) SAXS and (**b**) WAXS of 25GE (blue line), 25GE/DOX (red line) and 25GE/Crocin (black line) at 25 °C.

The WAXS profiles of the hydrogels reveal the diminishing of the amorphous region, likely due to its solubilization whilst remaining the refraction of the α-helices or the triple refraction that could indicate the renaturation of the single triple helix in good agreement with the FTIR spectra. Furthermore, the addition of drugs to the gelatin network (Figure 17a,b) slightly modifies the internal structure of the tuna hydrogel as shown by FTIR.

**Figure 18:**
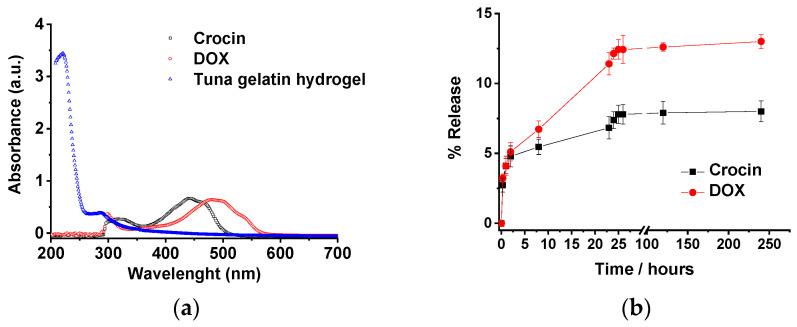
(**a**) UV-Vis spectra of non-loaded tuna gelatin hydrogel, Crocin and Doxorubicin in milli-Q water. (**b**) Release profiles of Crocin and DOX from loaded-gelatin at a different time in PBS pH 7.4 at 30 °C. The drug releases were tested in three replicates. Error bars are 2σ.

The revealed structure of the tuna hydrogels, as well as their properties (gel state and high viscosity at room temperature along with good mechanical stability), show the feasibility of using them as promising candidates for biomedical applications. In addition, the incorporation of the different hosting molecules by the gelatin hydrogel could be achieved both physically due to the topology of the small holes produced by the cross-linked networks as well as by non-covalent interactions such as hydrogen bond that allow the drug load by different methodologies that will determine the different release profiles. However, the sustained release in a biological medium of the drug remains a challenge for most of the hydrogel systems. Likewise, the release of both loaded antitumor drugs (Doxorubicin and Crocin) from the tuna hydrogel network was monitored by UV-Vis. Tuna hydrogel shows a typically main absorption band located at 215 nm together with a shoulder at 290 nm related to the presence of Type I collagen [62] whereas Free Crocin and DOX spectra present bands centered at 440 and 490 nm, respectively, that permits easily monitoring of the release profiles. The drug release profiles of tuna gelatin hydrogel in PBS buffer at 30 °C exhibit similar triphasic behavior for both drugs with a significant burst release during the first 5 h (≈5%), probably related to the drug molecules encapsulated in the gelatin pores as observed by cryo-SEM (Figure 18). Thereafter, two different pseudo-first-order release profiles were reached for both molecules, an initial faster release within the first 5 to 24 h and a slower release from 24 h onwards with different release rates for both antitumoral that could be associated with the disruption of physically cross-linked gelatin network [63]. The burst release is similar for both drugs, in agreement with the physical trap within the porous network. On the contrary, the slopes of pseudo-first older release are more pronounced for DOX which suggest that Crocin is more effectively trapped due to the hydrogen bond interactions as supported by FT-IR experiments. Besides, the release profile of DOX of the straightforward loaded tuna gelatin hydrogel was found to be similar to previously generated nanogels with a more sophisticated design approach [22].

## 3. Conclusions

Novel gelatin hydrogels based on the valorization of waste skin yellowfin tuna were easily generated as drug carrier vehicles for the delivery of antitumoral agents Doxorubicin and Crocin by local administration in complex human anatomical areas after surgical resection of cancer tissues and thus, minimizing the invasive detrimental effect on healthy tissues. According to molecular weight distribution and FTIR analyses, tuna gelatin was identified as a type I collagen which contains α helix, β sheet and random coil structures. The addition of the antitumoral (Doxorubicin and Crocin) suggested enhancing the organization of the gelatin structure using hydrogen bonds. Thermogravimetric analyses proved that gelatin hydrogel and drug-loaded gelatin hydrogels are sufficiently stable during the thermal treatment for the hydrogel formulations, even delaying the initial degradation step by the addition of the drugs. Morphological analyses of the formed hydrogels using cryo-SEM and AFM confirmed the small pore size of the generated hydrogel network which is useful to host the drug molecules and control the drug release. DSC and MDSC analyses of the hydrogels evidenced a single sol/gel transition at around 20 °C and a T_g_ at ca. −65 °C which is much lower than dry gelatin, confirming the plasticization by water, attributed to readjustments occurring in gelatin hydration. The mechanical properties exhibited by the hydrogels confirmed the elastic nature of the generated networks featuring a pronounced change in complex viscosity and storage modulus upon temperature increase related to the sol/gel transition as well as its reversibility upon cooling. The “syringe test” confirmed the feasibility of a hypothetical injection into the body of a specific diameter (0.9 mm) of the tuna hydrogel system at physiological conditions which is essential to ensure adequate intra-articular administration of gel-like systems. The drug delivery essay proved the good biocompatibility and high drug loading efficiency of the tuna gelatin hydrogels that due to their characteristic thermal and mechanical stability, multimodal capacity to host different molecules and chemoselectivity to control efficient release over time, they are excellent carriers for drug delivery applications by local administration methodologies.

## 4. Materials and Methods

### 4.1. Raw Materials

Fish skin waste (Y—yellowfin tuna, *T. albacares*) was kindly provided by Conservas Rianxeira S.A.U. (A Coruña, Spain). Fish skin gelatin (GE) extraction was performed as previously described [23]. Tuna (*T. albacares*) skin was selected due to its abundance as industrial processing waste.

For the therapeutic agents, Crocin was purchased from Sigma Aldrich and Doxorubicin (DOX) was purchased from MedChemExpress.

### 4.2. Methods

#### 4.2.1. Preparation of Gelatin Hydrogel

The tuna gelatin was weighed using a Mettler AE-240 electronic balance, with a precision of 5 × 10^−5^ g and then dispersed in a predetermined volume of ultrapure deionized water to obtain the gel composition of 25% weight fraction. Furthermore, the gelatin solution was stirred using a 5L Selecta low power ultrasonic bath (J.P. Selecta S.A., Barcelona, Spain) with an ultrasonic frequency of 40 kHz and an out power of 120 W for 30 min heating at 60 °C to disperse properly the fish collagen and subsequently cooling to room temperature (22 °C) forming a homogeneous gel.

#### 4.2.2. Preparation of Drug-Loaded Gelatin Hydrogel

Similarly, drug-loaded gelatin solutions were prepared by passive loading methodology. A corresponding amount of drug stock solution was added to the gelatin hydrogel to reach a final concentration of 1 mM, forming gelatin hydrogel/drugs.

#### 4.2.3. Gel Permeation Chromatography (GPC)

The molecular weight distributions of gelatin in solution were analyzed by gel permeation chromatography (GPC) using an Agilent 1260 HPLC equipped with a quaternary pump (G1311B), injector (G1329B), column oven (G1316A), diode array (G1315C), refractive index (G1362A) and double angle static light scattering detectors (G7800A). The sample was separated with four columns (Proteema, PSS, Mainz, Germany): precolumn (5 µm, 8 × 50 mm), 100 Å (5 µm, 8 × 300 mm), 300 Å (5 µm, 8 × 300 mm) and 1000 Å (5 µm, 8 × 300 mm). Elution was carried out at 20 °C with 0.15 M sodium acetate: 0.2 M acetic acid, pH 4.5 at 0.5 mL/min. The sample was dissolved at 2 g/L in the GPC mobile phase. The detectors were calibrated with a polyethylene oxide standard (PSS, Mainz, Germany) of 106 kDa (Mw) and a refractive index increment (dn/dc) of 0.135. For the gelatin sample, the molecular weights were calculated with a dn/dc of 0.190.

#### 4.2.4. Thermogravimetric Analysis (TGA)

The thermal stability of gelatin and gelatin hydrogel was determined using a Setsys Evolution 1750 thermogravimetric analysis instrument (TGA) (Setaram). A total of 5 mg of gelatin and 60 mg of tuna gelatin hydrogel were placed in a sealed capsule and subjected to temperature sweeps from room temperature to 800 °C, at a heating rate of 5 °C/min and under a nitrogen atmosphere. Experiments were conducted in triplicate. The deviations between the temperature transitions obtained from three different runs were lower than 0.5 K.

#### 4.2.5. Fourier Transform-Infrared Spectroscopy (FT-IR)

Attenuated Total Reflectance Fourier Transform Infrared Spectroscopy (ATR-FTIR) was used to study the effects of hydration on gelatin in terms of molecular and supramolecular structure and organization of gelatin secondary structure.

ATR-FTIR was obtained using a Nicolet 6700 spectrometer (Nicolet Instrument Corporation, USA) equipped with an IR -Turbo source and a DTGS detector. The spectra of the gelatin samples were obtained on a KBr beam splitter. The number of background scans was 34 and the tests were carried out at room temperature with a spectral resolution of 4 cm^−1^. The frequency range was 4000 to 400 cm^−1^. An attenuated total reflectance (ATR) fixture was used to measure the changes occurring in an internally reflected infrared beam when the beam comes into contact with the sample. The tuna gelatin and hydrogel were deposited on a gold support in a humid chamber to avoid evaporation during the experiments. The background was subtracted from sample scans. The integrated area of each peak was normalized to compare the spectra of both samples. The secondary structure was analyzed in more detail by the second derivative of the Amide I spectrum that was obtained using the First Difference Derivative (FDD method).

#### 4.2.6. Cryo-Scanning Electron Microscopy (Cryo-SEM)

The microstructures of the Tuna gelatin hydrogel at a concentration of 25% wt, were evaluated in cryo mode. The sample was mounted on the sample holder using a gold 13 mm sample holder, and frozen at −200 °C using liquid nitrogen. The sample was immediately transferred under liquid nitrogen in a cryogen box to the Baltec equipment (MODEL, MED-020). Then it was sliced on the surface (with a thickness of 2 to 3 mm). Finally, the fractured sample was visualized and photographed with a scanning electron microscope (JEOL JSM-6700) at an acceleration voltage of 5 kV.

#### 4.2.7. Atomic Force Microscopy (AFM)

The surface morphology of the Tuna gelatin hydrogel was also characterized using topographic characterization techniques, to complement the cryo-SEM analyses. Interferometric optical profilometry (WLOP) was conducted using a WYKO-Veeco NT 1100 while atomic force microscopy (AFM) was performed by Veeco Multimode 8 Nanoscope V.

Vertical scanning white light interferometry (VSI) mode was used for interferometric optical profilometry (WLOP). The samples were prepared by fixing them on an opaque sample holder, to enhance the reflection of light. The samples were fixed at the ends with an adhesive; so that they were as flat as possible without deforming them. For the (AFM), the aliquot is deposited on a silicon substrate.

WLOP provides us with measurements of larger fields of view (larger images), so we analyzed a wider area, obtaining an overview of the sample surface. On the other hand, AFM allows us to measure smaller fields with greater precision and resolve the microstructure or structure with higher resolution.

Scanning Probe Microscopy Multimode 8/Nanoscope V (AFM-STM) is a mechano-optical instrument that detects forces at the atomic level (in the order of nano Newtons) through the optical measurement of the movement on the Surface of a cantilever. The AFM averages have been made by two methods; Tapping Mode and Peak, and Force Tapping Mode, to analyze which method best solved the microstructure and obtained greater clarity in the image. The best results were obtained with the Tapping Mode, therefore the results were focused on the images made by Tapping Mode. To calculate the three-dimensional roughness parameters (Sa, Sq, Sz, Ssk and Sku), a “Cylinder and Tilt” correction was applied beforehand to eliminate plane inclination and minimize the influence of geometric deformations. The calculations were carried out without applying interpolation in the few missing areas of pixels; however, to represent the images and improve their visual quality, the interpolation “Data Restore” was applied.

#### 4.2.8. Rheology

The consistency of the gelatin hydrogel (gel point, viscosity, and viscoelasticity) is critical to understanding the strength of the gel which are also especially important for biomedical applications.

The rheological properties of the tuna gelatin hydrogel have been determined using a Physica MCR 101 rheometer (Anton Paar, Graz, Austria). The temperature was controlled by a Peltier P-PTD 200, placed in the bottom plate [64]. The cone-plate geometry was employed for strain and frequency sweep measurements, while plate-plate geometry was for temperature ramp measurements. Strain and sweep frequency sweeps were performed using a cone-plate geometry (CP 50-1), with a constant space of 0.102 mm whilst the resistant plate-plate (PP50/S) geometry was selected to conduct temperature sweeps at constant strain and frequency with a geometry gap of 0.1 mm to control the torque between 0.5 μN·m and 125 μN·m [64]. Experiments were repeated three times to analyze the reproducibility of obtained results.

The linear viscoelastic range was determined previously by performing a strain sweep from 0.1 to 1000% at a constant angular frequency of 10 rad/s at 20 °C. The storage modulus G′ and the loss modulus G″ were determined in the linear strain range using a constant 0.1% strain. Frequency sweep measurements were made from 0.05 to 100 rad/s applying a constant 0.1% strain at 20 °C. The gelation of the gelatin hydrogel was studied by temperature ramps, in a temperature range of 10 to 40 °C for the heating ramps and a range of 40 to 10 °C for the cooling ramps.

Oscillatory (dynamic) rheological studies were performed to probe the sol-gel (gelation point) and gel-sol (breakdown of the gel network) transitions as well as the viscoelastic character of the final gel structure. Three types of dynamic rheological experiments were performed to examine the gel network during gelation and the limits at which the network will break. The solutions were preheated to 60 °C for 30 min for all rheological experiments to disrupt the gel network.

The syringe tests were performed using a plate geometry (PP25/S) with a plate diameter of 25 mm and a geometric gap of 0.1 mm. A three-step experiment based on literature [65] was performed to evaluate the response of tuna gelatin hydrogel to simulated injection into the human body. Firstly, a resting state of the material in the syringe was simulated, by applying a strain of 0.1% and a frequency of 1 Hz, at room temperature. Subsequently, the temperature was increased to 37 °C at a constant shear rate of 100 s^−1^, mimicking an injection using a 0.9 mm diameter needle (commonly selected for the administration of these formulations). Finally, a strain of 0.1% was reimposed in the linear viscoelasticity range and a frequency of 1Hz at a temperature of 37 °C, simulating the resting state of gelatin hydrogel in the human body. Deviations in (complex) viscosities between repetitions were lower than 3%.

#### 4.2.9. Differential Scanning Calorimetry (DSC)

The glass transition (Tg) and other thermal parameters of dry GE, 25GE, 25GE/DOX and 25GE/Cro were determined employing a Q2000 differential scanning calorimeter, (DSC) (TA Instruments, New Castle, DE, USA) equipped with a refrigerated cooling system RSC90. About 80 mg of sample was placed and hermetically crimped in high-volume stainless-steel cells with rubber O-ring seal able to withstand pressures up to 10 MPa. The DSC chamber was purged with nitrogen (molar fraction purity > 0.99999) at a flow rate of 50 mL·min^−1^. Standard DSC thermograms were obtained heating/cooling at 1 and 5 °C·min^−1^ in the temperature range between −80 °C and 200 °C (in the case of the dry GE) and −80 °C and 100 °C (for the hydrogels). Modulated differential scanning calorimetry (MDSC) analyses were performed at 0.5 °C·min^−1^, with a modulation amplitude of ±0.08 °C, a modulated period of 60 s. Scanned temperature ranges in MDSC tests were the same as in runs done in standard mode. Before each run, the sample was equilibrated for 5 min at the initial temperature. Glass transition temperatures were estimated as the midpoint of the line drawn between the temperature at the intersection of the initial tangent with the tangent through the inflexion point of the trace and the temperature of the intersection of the tangent through the inflexion point with the final tangent. The reported experimental values are based on at least three different replicates. The deviations between the temperature transitions obtained from the different runs were lower than 0.3 K.

#### 4.2.10. X-ray Scattering

Small-angle X-ray scattering and wide-angle X-ray experiments were performed in NCD-SWEET beamline at ALBA synchrotron facilities, (Cerdanyola del Vallès, Spain) using a wavelength of 1 Å: 12 keV and a spot size of 100 nm × 100 nm (FWHM). A Pilatus 1 M detector with a sensitive area of 981 × 1043 pixels of 172 µm × 172 µm was used as SAXS detector at a sample to detector distance of ca. 2.5 m. A Rayonix (LX255-HS) with an active area of 1472 × 195 pixels with a pixel size of 172 µm × 172 µm was placed at a distance of ca. 0.31 m for collecting the WAXS patterns. The scattering patterns of Silver behenate (AgBe) and α-alumina (α-Al_2_O_3_) were employed to calibrate the scattering vector q by using the well-known diffraction reflections positions where q = 4πsinθ/λ with θ being half of the scattering angle. Background subtraction amended by X-ray transmission through the sample was applied to every 2D pattern normalized upon the incident intensity of the frame recorded to avoid beam fluctuations. The 2D scattering corrected patterns (SAXS and WAXS) were reduced to 1D profiles of scattered intensity in arbitrary units as a function of the scattering vector using the Bubble [66] free software.

The heated hydrogel solutions were introduced in a glass capillary of 1.5 mm (diameter) and sealed before being introduced in the Linkam heating stage, while the dry tuna gelatin was evaluated introduced in a holder fitted with Kapton windows. The measurements were carried out at 25 °C.

The experimental SAXS data were fitted by an in-house program [67,68] that uses the minimization MINUIT program created by CERN [67,68]. The measured SAXS profile intensity (I(q)) were adjusted to an interacting disk-like shape model [69] described by a structure factor of hard-disk mixtures resolved by an analytic theory resulting from a free-energy functional for the inhomogeneous hard-disk fluid [70]. The pores observed by cryo-SEM suggested a random uniform distribution that was previously modelled [71] with the hard-disk structure factor (Equations (2) and (3)) [63] to consider the scattering interference aroused from the different pores by interpolating the analytical solution of a two-dimensional mixture of inhomogeneous discs from the analytical cases of one and three dimensions and with the structure factor defined as:

(1) $$I\;\left(Q\right)=\rho_{0}\upsilon^{2}\frac{2}{Q^{2}R^{2}}\left(1-\frac{J_{1}\left(2QR\right)}{QR}\right)$$

(2) $$\frac{1}{S\left(q\right)}-1=4\eta \left\{\;A{\left[\frac{J_{1}\left(qR\right)}{qR}\right]}^{2}+B\cdot J_{0}\left(qR\right)\cdot \frac{J_{1}\left(qR\right)}{qR}+G\cdot \frac{J_{1}\left(2qR\right)}{qR}\right\}$$

(3) $$\begin{array}{lll} A & = & \left[1+\left(2\eta -1\right)\chi +2\eta G\right]/\eta \\ B & = & \left[(1-\eta \right)\chi -1-3\eta G]/\eta \\ Z & = & 1/{\left(1-\eta \right)}^{2}\\ \chi & = & \left(1+\eta \right)/{\left(1-\eta \right)}^{3}\\ G & = & 1/\left(1-\eta \right) \end{array}$$

#### 4.2.11. Drug Release Methodology

Drug release experiments. The amount of drug released from the gelatin was determined through incubation at 30 °C of 200 μL of each drug-loaded gelatin. Then, 3 mL of PBS buffer at physiologic PBS pH 7.4 were added to drug-loaded gelatin. The simultaneous degradation of the gelatin and the release of the drug were visually observed. The drug release profile was examined by recording UV-Vis spectra for 10 days. The drug-release concentration was measured at 470 and 500 nm for Crocin and DOX, respectively, using PBS as reference. The drug releases were tested in three replicates. The drug release profiles are based on at least three independent measurements.

UV-Vis studies. The UV-Vis absorption spectra were recorded using a Cary 100 (Varian) in a dual-beam spectrophotometer in a 10 mm quartz cuvette, with a step resolution of 1 nm at room temperature. A quartz cuvette (Hellma Analytics) fitted with a 1 cm optical path was used for the measurements. The device is equipped with a temperature-controlled cuvette holder, TLC 50 (Quantum Northwest). For the UV-Vis spectra of non-loaded gelatin, samples were prepared directly in the quartz cuvette following the procedure described above. UV-Vis spectra of non-loaded gelatin, DOX and Crocin were measured in milli-Q water as a reference.

## Figures and Tables

**Table 1:** Correspondence between the abbreviated code and full naming code (description of gelatins) under study.

Abbreviated Name	Full Name (Mass Compositions of Gelatin and Drugs)
Dry GE	Dry Tuna
25GE	25% Tuna gelatin hydrogel in water
25GE/DOX	25% Tuna gelatin hydrogel loaded/1 mM DOX in water
25GE/Crocin	25% Tuna gelatin hydrogel loaded/1 mM Crocin in water

**Table 2:** Molecular weight of gelatin from tuna skin of the distributions shown in Figure 2 (mean ± standard deviation; *n* = 2). Rt: retention time; Mw: weight average molecular weight. Peak area (%) corresponds to the refractive index detector.

	Peak Number	Rt (min)	Mw (kDa)	Peak Area (%)	Possible Structure Elements
Dry GE	1-high Mw	38.0–44.3	>300 kDa	19.8	γ-/other HMw aggregates
	2	45.4	228.0 ± 9.9	8.2	β-chains
	3	46.0	156.5 ± 14.7	19.5	Degradated β-chains or aggregates of α-chains with other peptides
	4	48.3	117.0 ± 7.6	15.4	α-chains
	5-low Mw	49.3–60.0	<100	37.1	degraded peptides

**Table 3:** Values obtained from TGA and DTGA analyses of Dry GE, 25GE, 25GE/DOX and 25GE/Crocin. Onset temperatures, T_onset_; maximum temperatures, T_max_; and weight loss (%) at 5 °C/min.

	Steps	T_Onset_ (°C)	T_max_ (°C)	Weight Loss (%)
Dry GE	1	19	182	−11
	2	258	323	−62
	3	614	650	−25
25GE	1	70	90	−71
	2	106	117	
	3	245	311	−16
25GE/DOX	1	63	101	−78
	2	257	312	−12
25GE/Crocin	1	96	117	−81
	2	244	323	−11

**Table 4:** Weight loss reason at the maximum peaks of temperature shown in Table 3.

Steps (°C)	Samples	Weight Loss Reason
Step 181 °C	Dry GE	- Decomposition of polysaccharide/protein components of the gelatin - Evaporation of the adsorbed and bound water
Step 90–115 °C	25GE 25GE/Crocin 25GE/DOX	- Loss of free water adsorbed on the gelatin structure
Step 310–325 °C	Dry GE 25GE 25GE/Crocin 25GE/DOX	- Breakdown of the gelatin peptide bonds due to the degradation of the low molecular weight protein fraction - Structurally bound water
Step 600 °C	Dry GE	- Thermal decomposition of the gelatin networks due to the formation of covalent bonds in the gelatin network

**Table 5:** Assignments of IR bands to the secondary structure of Dry GE, 25GE, 25GE/DOX and 25GE/Crocin.

Secondary Structure Elements	Wavenumber (cm^−1^)			
	Dry GE	25GE	25GE/DOX	25GE/Crocin
B-Turn	1682	1674 1682	1674 1682	1674 1682
B-Sheet	1624 1632	1624 1634	1626 1635	1624 1632
Triple Helix	1649 1660	1651 1660	1651 1660	1650 1659
3_10_ Helix	1670	1668	1668	1668
Random Coil	1643	1645	1645	1643

**Table 6:** 2D Roughness parameters were calculated from the 1 × 1 µm^2^ field of view images obtained by AFM of Zone 1, Zone 2 and Zone 3.

AFM/2D Amplitude Roughness Parameters/Imaging 1.1 × 1.1 µm^2^					
	Ra (nm)	Rq (nm)	Rz (nm)	Skewness	Kurtosis
Zone 1	0.242	0.304	2.56	0.049	3.00
Zone 2	0.282	0.353	4.31	0.147	3.12
Zone 3	0.292	0.366	3.46	0.095	3.06
Average	0.27	0.34	3.44	0.10	3.06
Standard deviation	0.03	0.03	0.88	0.05	0.06

**Table 7:** Glass transition temperature (Tg), and enthalpy of melting (ΔHm) of Dry GE and 25GE in the Non Reversing thermogram (MDSC).

Type of Gelatin	Tg (°C)	ΔHm (J/g)
Dry Tuna	68.9	2908
25GE	−65	NA

## Data Availability

Not applicable.

## Supplementary Materials

The following supporting information can be downloaded at: https://www.mdpi.com/article/10.3390/gels8040237/s1, Figure S1: (a) Comparison of FTIR spectra at room temperature (dashed lines) and FTIR spectra at 50 °C (solid lines) in the 1600–1700 cm^−1^ region, (b) Comparison of FTIR spectra at room temperature (dashes lines) and FTIR spectra at 50 °C (solid lines) in the 2900–4000 cm^−1^ region, of (-) Dry GE, (-) 25GE, (-) 25GE/DOX and (-) 25GE/Crocin; Figure S2: (a) DSC thermogram of Dry GE at 5 °C·min^−1^ (orange color) and at 1 °C·min^−1^ (green color), (b) DSC thermogram of 25GE at 1 °C·min^−1^; Figure S3: MDSC of (a) Dry GE in the Rev Heat Flow (green colour), Non Rev. Heat Flow (Black colour) and Heat Flow (red colour), (b) Dry GE at 1 K/min (Two reps), (c) 25GE in the Rev Heat Flow (green colour), Non Rev. Heat Flow (Black colour) and Heat Flow (red colour), (d) MDSC of 25GE in the Reversing Heat Flow; Table S1: The compilation of the major band assignments for Dry GE, 25GE, 25GE/Dox, and 25GE/Crocin at 25 °C; Table S2. Major band assignments for Dry GE, 25GE, 25GE/Dox, and 25GE/Crocin at 50 °C.
