# Supplementary material for: Characterization of Tuna Gelatin-Based Hydrogels as a Matrix for Drug Delivery
Source: Gels. 2022 Apr 12;8(4):237. doi: 10.3390/gels8040237 (PMC9026235; doi:10.3390/gels8040237)
Supplement: Supplementary file 1 [file gels-08-00237-s001.zip › gels-1601599-supplementary.pdf]

SUPPLEMENTARY INFORMATION

Characterization of Tuna Gelatin-Based Hydrogels as a Matrix for Drug Delivery

Carolina Hermida-Merino[1,*], David Cabaleiro[1], Luis Lugo[1], Jesus Valcarcel[2], Jose A. Vázquez[2], Ivan Bravo[3], Alessandro Longo[4,5], George Salloum-Abou-Jaoude[6], Eduardo Solano[7], Carlos Gracia-Fernández[8], Manuel M. Piñeiro[1], Daniel Hermida-Merino[1,9*]

Table S1. The compilation of the major band assignments for Dry GE, 25GE, 25GE/Dox, and 25GE/Crocin at 25°C.

Wavenumber (cm^{-1})				Assignment
**Dry GE**	**25 GE**	**25 GE/Dox**	**25 GE/Crocin**	
3284	3330	3297	3297	N-H/O-H
2850-3000	2850-3000	-	-	Aliphatic bond stretching CH
1333, 1444	1338, 1462	-	-	Aliphatic bending CH
1631,1528,1238	1634,1557,1244	1637, 1555, 1247	1635, 1558, 1248	Amide I,II,III
975	-	973		CNC
528,612,700	532,607,704	-	-	Amide IV,V,VI
1164-1080	1165,1082,1032	1200-1011	1200-1028	Bond stretching C-O, C-O-C
600	600	652	654	Water
-	-	621	-	N-C=O

Table S2. Major band assignments for Dry GE, 25GE, 25GE/Dox, and 25GE/Crocin at 50°C.

Wavenumber (cm^{-1})				Assignment
**Dry GE**	**25 GE**	**25 GE/Dox**	**25 GE/Crocin**	
3284	3280	3285	3285	N-H/O-H
2850-3000	2850 - 3000	2890 - 3000	2890 - 3000	Aliphatic bond stretching CH
1334, 1447	1334, 1447	1300-1447	1335-1447	Aliphatic bending CH
1633, 1519, 1236	1627, 1530, 1239	1630, 1529, 1237	1630, 1529, 1237	Amide I,II,III
920	-	973	973	CNC
561, 660, 703	556, 657, 704	-	-	Amide IV,V,VI
1163, 1080, 1031	1164, 1080, 1032	1200-1031	-	Bond stretching C-O, C-O-C
600	600	652	-	Water
-	-	621	-	N-C=O
-	-	1730	-	C=O from Dox

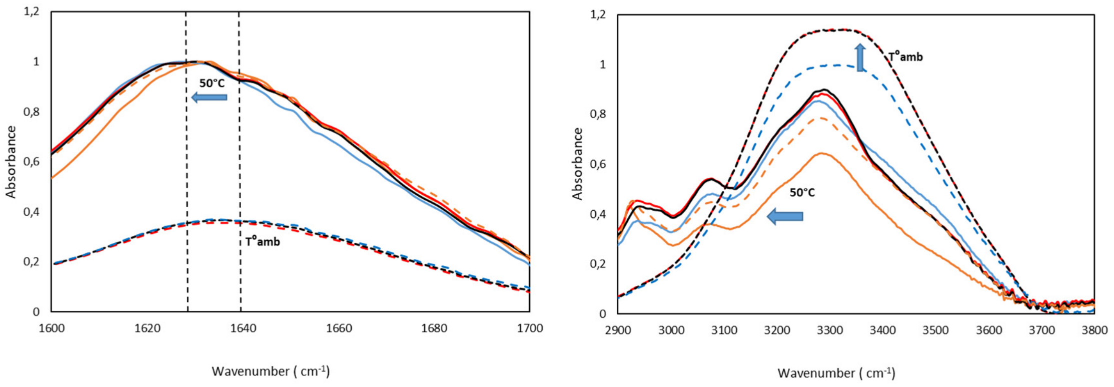

(a) (b)

Figure S1. (a) Comparison of FTIR spectra at room temperature (dashed lines) and FTIR spectra at 50°C (solid lines) in the 1600-1700 cm^{-1} region, **(b)** Comparison of FTIR spectra at room temperature (dashes lines) and FTIR spectra at 50°C (solid lines) in the 2900-4000 cm^{-1} region, of **(-)** Dry GE , **(-)** 25GE, **(-)** 25GE/DOX and **(-)** 25GE/Crocin.

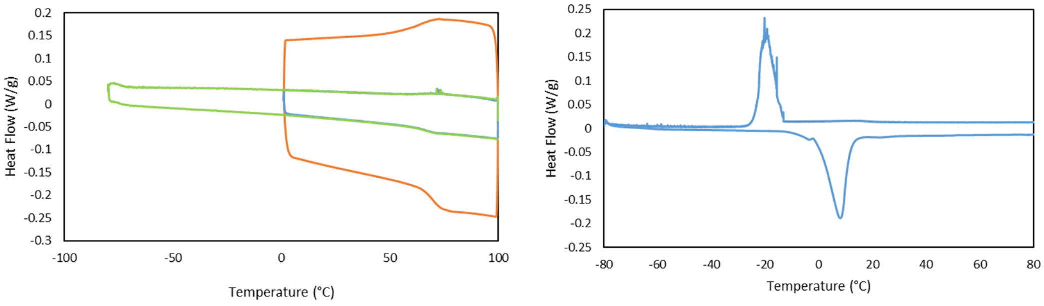

(a) (b)

Figure S2. (a) DSC thermogram of Dry GE at 5°C·min^{-1} (orange colour) and 1°C·min^{-1} (green colour), **(b)** DSC thermogram of 25GE at 1°C·min^{-1}.

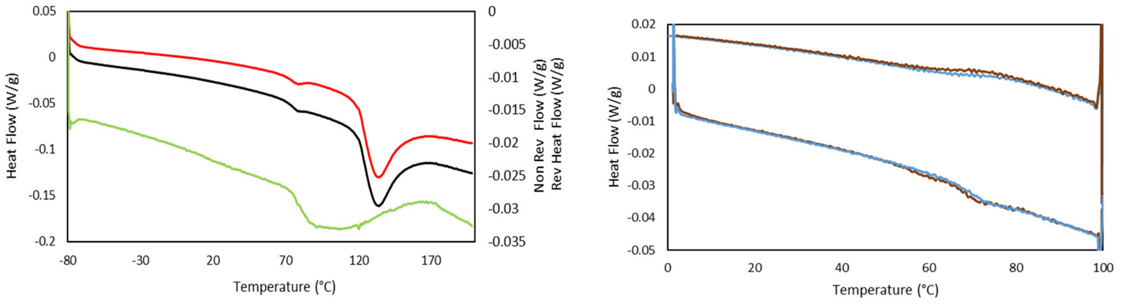

(a) (b)

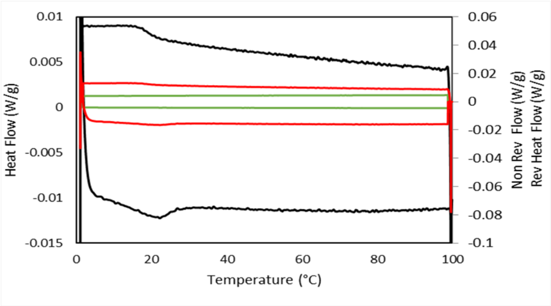

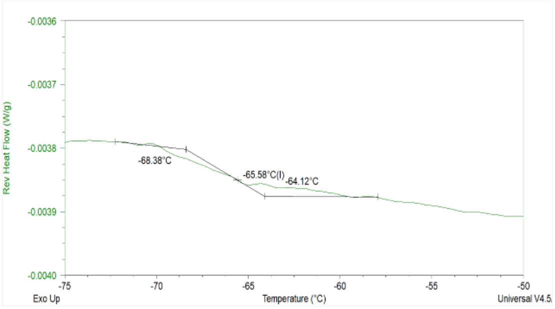

c) **d)**

Figure S3. MDSC of **(a)** Dry GE in the Rev Heat Flow (green colour), Non Rev. Heat Flow (Black colour) and Heat Flow (red colour), **(b)** Dry GE at 1K/min (Two reps), **(c)** 25GE in the Rev Heat Flow (green colour), Non Rev. Heat Flow (Black colour) and Heat Flow (red colour), **(d)** MDSC of 25GE in the Reversing Heat Flow.
